# Legal abortion in childhood: the official discourse and the reality of a Brazilian case

**DOI:** 10.1590/0034-7167-2021-0946

**Published:** 2022-07-18

**Authors:** Lucimara Fabiana Fornari, Emiko Yoshikawa Egry, Mariana Sbeghen Menegatti, Karen Namie Sakata So, Rosa Maria Godoy Serpa da Fonseca, Maria Amélia de Campos Oliveira

**Affiliations:** I Universidade de São Paulo. São Paulo, São Paulo, Brazil

**Keywords:** Child Abuse, Abortion Legal, Reproductive Rights, Sex Offenses, Public Policy., Maltrato a los Niños, Aborto Legal, Derechos Sexuales y Reproductivos, Delitos Sexuales, Política Pública., Maus-tratos Infantis, Aborto Legal, Direitos Sexuais e Reprodutivos, Delitos Sexuais, Política Pública.

## Abstract

**Objective:**

to identify the ideological perspectives of official discourses in relation to sexual violence, childhood pregnancy and access to legal abortion based on a Brazilian case.

**Methods:**

a qualitative documentary study. Data collection was carried out in documents published on official Brazilian websites, between August and December 2020. The analytical categories of gender and generation supported data analysis.

**Results:**

a total of 39 documents were selected and three empirical categories were identified: Protection against violence in the legislation and the (re)production of injuries in reality; Facing sexual violence against children by the Brazilian State; Being a Brazilian girl: gender and generational oppressions.

**Final considerations:**

the ideological perspectives of official discourses in relation to the case showed a lack of compliance with advances in Brazilian legislation on issues related to child violence and adult-centric authoritarianism, with the imposition of gender and generation subalternity.

## RESUMO

**Objetivo:**

identificar as perspectivas ideológicas dos discursos oficiais em relação à violência sexual, à gravidez na infância e ao acesso ao aborto legal a partir de um caso brasileiro.

**Métodos:**

estudo documental qualitativo. A coleta de dados foi realizada em documentos publicados em sites oficiais brasileiros, entre agosto e dezembro de 2020. As categorias analíticas de gênero e geração deram suporte à análise dos dados.

**Resultados:**

foram selecionados 39 documentos e identificadas três categorias empíricas: *A proteção contra a violência na legislação e a (re)produção dos agravos na realidade; O enfrentamento da violência sexual contra a criança pelo Estado brasileiro; Ser menina brasileira: opressões de gênero e geração*.

**Considerações finais:**

as perspectivas ideológicas dos discursos oficiais em relação ao caso mostraram falta de adesão aos avanços na legislação brasileira nos temas relativos à violência infantil e autoritarismo adultocêntrico, com imposição de subalternidade de gênero e geração.

## RESUMEN

**Objetivo:**

identificar las perspectivas ideológicas de los discursos oficiales en relación a la violencia sexual, el embarazo infantil y el acceso al aborto legal a partir de un caso brasileño.

**Métodos:**

estudio documental cualitativo. La recolección de datos se realizó en documentos publicados en sitios web oficiales brasileños, entre agosto y diciembre de 2020. Las categorías analíticas de género y generación apoyaron el análisis de datos.

**Resultados:**

se seleccionaron 39 documentos y se identificaron tres categorías empíricas: Protección contra la violencia en la legislación y la (re)producción de lesiones en la realidad; Enfrentando la violencia sexual contra los niños por parte del Estado brasileño; Ser niña brasileña: opresiones generacionales y de género.

**Consideraciones finales:**

las perspectivas ideológicas de los discursos oficiales en relación al caso mostraron una falta de adhesión a los avances de la legislación brasileña en temas relacionados con la violencia infantil y el autoritarismo adultocéntrico, con la imposición de la subalternidad de género y generacional.

## INTRODUCTION

Children are vulnerable to different types of violations, such as physical, psychological, sexual, institutional, structural, intrafamilial and moral, in addition to neglect, child labor and denial of rights. Sexual violence and the attempt to prevent abortion were two of the various violations filed against Violeta, a fictitious name given to a victim of sexual violence in a Brazilian case that will be the subject of analysis in this research.

The Brazilian Federal Constitution establishes that the family, society and State must guarantee children’s fundamental rights and protection against different forms of violations^(1)^. Among the advances in Brazilian legislation related to childhood, an important legal framework was the Child and Adolescent Statute (ECA - *Estatuto da Criança e do Adolescente*), considered the main normative and regulatory instrument of rights. ECA states that no child or adolescent shall be the object of any form of negligence, discrimination, exploitation, violence, cruelty or oppression^(2)^.

Although children’s rights are guaranteed by Brazilian law, in 2020, the year ECA turned 30, an emblematic case of violence against children was recorded and widely discussed in the Brazilian media. Violeta, 10 years old, living in São Mateus, Espírito Santo, sought care at the *Hospital Universitário Cassiano Antônio de Moraes* of the *Universidade Federal do Espírito Santo*, accompanied by her grandmother, complaining of abdominal pain. After medical assessment, a pregnancy of 22 weeks was found, a result of rapes perpetrated by her uncle since the age of six^(3)^.

With the case disclosure by the media, Violeta was the target of an intense campaign against abortion by religious and political groups, had her identity exposed on social media, was labeled a murderer and faced difficulties in accessing legal abortion due to the refusal of the health establishment where she was initially treated. Finally, with judicial authorization, the termination of pregnancy was performed by another reference health service, the *Centro Integrado de Saúde Amaury de Medeiros* ^(3)^, located in Recife, Pernambuco.

This case highlights the harsh reality of Brazilian girls and women, victims of sexual violence, often perpetrated by acquaintance, which have as a consequence pregnancy. Forced child pregnancy is considered when a girl under the age of 14 becomes pregnant without consent and has access to legal abortion hampered or denied. Generally, when child pregnancy becomes known, different opinions about the conduct to be taken are observed disregarding the victim’s voice and will. These opinions commonly refer to continuity of pregnancy, even if this poses a risk to health and life itself^(4)^.

Pregnancy in girls under the age of 14 is considered a crime because, in most cases, it is the result of sexual violence. This type of violation is often accentuated by other types of violence, perpetrated through gender stereotypes, health care and the obstacles of legislation to stop pregnancy^(5)^.

A scoping review of the legal responsibility of low- and middle-income countries in sexual and reproductive health found that studies related to abortion highlighted the need to implement legislation as a guide to practice of care. Among the reasons for non-implementation were lack of knowledge by stakeholders, ideological opposition of community members or professionals, and practice of conscientious objection^(6)^.

In Brazil, according to the Hospital Information System, in 2020, 86 abortions were recorded for medical reasons in girls, aged 10 to 14 years. In 2021, until September, 102 similar abortions were recorded^(7)^. Unfortunately, this data reveals only a portion of the phenomenon, because it concerns those girls who managed to have access to legal abortion. Another portion continues pregnancy, seeks clandestine services to perform abortion or becomes a victim of maternal mortality. It is noteworthy that, between 2015 and 2019, 68 deaths of Brazilian women aged 10 and 14 years were recorded due to pregnancy^(8)^.

How many of these deaths would be consequences of this situation? It is impossible to say. The available data can hide the magnitude of the problem due to the complexity and taboos surrounding child pregnancy. Moreover, a search carried out by the authors in national and international databases found a scarcity of studies on the subject, as publications that use the term early pregnancy also refer to the period of adolescence. However, it is important to consider this generalization, because being a mother at the age of 10 has greater unfavorable implications than at the age of 17.

In Brazil, the exact number of girls under the age of 14 who become pregnant due to sexual violence is not known. This lack of information about the overview of child pregnancy and access to legal abortion arouses attention about how the State, represented by public agents, is ideologically positioned in relation to coping with the problem, which justifies the development of this study. Based on the above, this research had as a fundamental question: what are the ideological perspectives present in the official discourses regarding the case of sexual violence and the interruption of Violet’s pregnancy?

It is worth clarifying that in this research ideology is understood as:

A logical, systematic and coherent set of representations (ideas and values) and norms or rules (of conduct) that indicate and prescribe to the members of society what they should think and how they should think, what they should value and how they should value it, what they should feel and how they should feel, what they should do and how they should do. It is, therefore, an explanatory body (representations) and practical (norms, rules, precepts) of a prescriptive, normative, regulatory nature, whose function is to give the members of a class-divided society a rational explanation for social, political and cultural differences, without ever attributing such differences to the division of society into classes, starting from divisions in the sphere of production^(9).^

## OBJECTIVE

To identify the ideological perspectives of official discourses in relation to sexual violence, childhood pregnancy and access to legal abortion based on a Brazilian case.

## METHODS

### Ethical aspects

The research did not require the approval by Research Ethics Committee, as it used documents that were publicly available and freely accessible. The anonymity of data sources was guaranteed by replacing the name with the expression “Doc”, followed by Arabic numerals. The COnsolidated criteria for REporting Qualitative research (COREQ) instrument was used to describe methodological procedures.

### Study design and theoretical-methodological framework

This is documentary research, with a qualitative approach, which had the Theory of Praxic Intervention in Nursing in Collective Health (TIPESC) as a theoretical-methodological framework. Founded on the socially determined health process’s conception, TIPESC is a nursing theory that aims at intervention in objective reality and is based on the historical and dialectical materialist world view for analysis and understanding of phenomena in their dimensions: singular (of individuals and their families), particular (of groups and social strata) and structural (more general of society)^(10)^.

### Data source

The empirical material consisted of documents published on Brazilian official websites that addressed sexual violence, child pregnancy and access to legal abortion, in Violeta’s specific case.

Pronouncements, decrees, norms and legislation authored or emanating from federal, State and local (hospitals) representatives, published by official online pages, related to the themes of sexual violence, child pregnancy and legal abortion, referring to Violeta, were included.

### Data collection and organization

Data were collected between August 7 and December 1, 2020. The date of initiation of collection coincides with the day on which pregnancy was diagnosed by the health service. Closure was determined when the searches did not find new publications related to the case, in a period of 30 days.

The terms sexual violence, rape, pregnancy and abortion were used to search the documents on the official websites. Websites of the federal government, national councils and institutions that provided direct assistance to Violeta were accessed: Ministry of Women, Family and Human Rights (MWFHR); Ministry of Health (MoH); Civil House; Brazilian National Health Council (CNS - *Conselho Nacional de Saúde*); Brazilian National Council for the Rights of Children and Adolescents (CONANDA - *Conselho Nacional dos Direitos da Criança e do Adolescente*); Government of Espírito Santo; Government of Pernambuco; *Hospital Universitário Cassiano Antônio Moraes; Centro Integrado de Saúde Amaury de Medeiros*.

Twenty-five documents published by CONANDA, 11 by the MWFHR, five by the CNS, three by the MoH, two by the Government of Espírito Santo and documents published by the Civil House, Government of Pernambuco, *Hospital Universitário Cassiano Antônio Moraes* and *Centro Integrado de Saúde Amaury de Medeiros* (one of each) were selected. Two references identified in the selected documents were also included, totaling 52 publications.

### Data analysis

After reading in full and applying the inclusion criteria, 39 publications were selected for analysis. The texts were saved as Portable Document Format (PDF). Data were extracted using an instrument adapted to an Excel spreadsheet, developed by the authors, for the exclusive use of this research, aiming at identifying place and year of publication, type of document and discourses about gender, generation, sexual violence, child pregnancy and abortion.

The texts were submitted to thematic content analysis, consisting of pre-analysis, material exploration, treatment of results, interpretation and inference^(11)^, with the support of webQDA^(12)^. In the software, an Excel spreadsheet was inserted into the internal font system through automatic import. In the coding system, data related to the characterization of documents (place of publication, year and type of document) were automatically coded using descriptors. The empirical categories resulting from the analysis were constructed through tree codes.

The construction of empirical categories was carried out in the light of gender and generation analytical categories. The gender category considers inequalities that come from differences between men and women in society, constituting power relations, and the meanings attributed to the different ways of expression of masculinity and femininity^(13)^. The generation category is a social construction created from various parameters of society, whether historical, economic, political, cultural and others. In this understanding of the generational category, childhood refers to the place occupied by children in the structure of society, which has the adult category as hegemonic and dominant^(14)^.

## RESULTS

Of the 39 documents analyzed, 24 were published in 2020. The others were published in previous years, as they legally supported and influenced the outcome of the case; the first was ECA, from 1990. As for the types of publications, notes (8), news (6), resolutions (6), laws (3), manifestos (2), recommendations (2), decrees (2), letters (2) and reports (2) stand out. A letter, a decision by the Brazilian National Justice Department, a national policy, a national plan, an ordinance and a base document of the XI Brazilian National Conference on Children’s and Adolescents’ Rights were also found.

Data analysis allowed us to identify three empirical categories: *Protection against violence in the legislation and the (re)production of injuries in reality; Facing sexual violence against children by the Brazilian State; Being a Brazilian girl: gender and generational oppressions*.

### Protection against violence in the legislation and the (re)production of injuries in reality

In Brazil, children’s rights are guaranteed by the Federal Constitution, the Penal Code and ECA, documents in which they are responsible for guaranteeing these rights to the family, society and the State, represented by the Union, Federal District, states and cities.

*Art. 227. It is the duty of the family, society and the State to ensure children, adolescents and young people, with absolute priority, the right to life, health, food, education, leisure, professionalization, culture, dignity, respect, freedom and family life. and community, as well as protecting them from all forms of neglect, discrimination, exploitation, violence, cruelty and oppression.* (Doc 8)

The official documents also mentioned the services responsible for child welcoming and care, with emphasis on the family protection network, the justice system, public security, the health system and councils. However, in the case of Violeta, the lack of accountability of the Guardianship Council in monitoring the intersectoral network was evidenced.

(...) *the need for greater action by the Guardianship Council in the face of child protection actions was observed, including the dialogue between the child and her family with the Judiciary and the Public Ministry. This function is primarily of the Guardianship Council.* (Doc 7)

In addition to the deficient performance, the case reveals the curtailment of legally guaranteed rights, identified by the lack of protection against sexual violence and by the care conducted according to adults’ will, represented by government agents and professionals from the intersectoral network.

*We are concerned and indignant about the pilgrimage of a 10-year-old child, a victim of rape since the age of 6, who had to travel from one state to another in search of this public health service, which he was denied at first. Legal abortion, guaranteed by Brazilian legislation in cases of rape, must be accessed by all women who need it, at the appropriate time and place.* (Doc 19)

*The child was welcomed by the multidisciplinary team of the Program for Assistance to Women Victims of Sexual Violence, which has been operating at the University Hospital since 1998. After examinations, the team found that the conditions of pregnancy development (22 weeks and four days and a fetal weight of 537 grams) indicated a procedure that does not fit the protocol adopted by the hospital.* (Doc 13)

The violence experienced by Violeta did not only occur in the singular sphere, when she suffered successive violations by her uncle, but also when she sought support from the intersectoral network, since a portion of professionals was vehemently opposed to abortion.

*They irresponsibly used the pain of a child and a family in favor of ideological banners that do nothing to improve the mechanisms for protecting children.* (Doc 2)

*Even more revolting is to witness the involvement of public agents, responsible for the execution of protection, defense and reception laws, acting contrary to what is imputed to them by office. Religious fundamentalism is incapable of a democratic State of law, which in its Citizen Constitution expresses that Brazil is a secular State.* (Doc 19)

The position against abortion expressed by a portion of professionals in the intersectoral network and public agents was influenced by an ideological perspective based on religious fundamentalism. This reveals that, in practice, personal conjectures were superimposed on the rights provided by Brazilian legislation.

### Facing sexual violence against children by the Brazilian State

Official documents consider sexual violence a complex phenomenon based on gender and generation inequities, without presenting how these two social categories are understood. They recognize that this type of violence triggers reflexes for child development and traumatic consequences in adult life. The occurrence of pregnancy is an aggravating factor of the violence experienced, as can be seen in the following excerpts.

*This form of* [sexual] *violence is revealed in unequal power relations between children, adolescents and adults, and is permeated by the socioeconomic and gender inequalities present in society. In addition, it demonstrates the permissiveness of society in relation to objectification of the female body, the early eroticization of girls and what has been called rape culture.* (Doc 35)

*Violence creates scars and generates serious consequences in the biopsychosocial development of victims, and can produce traumas that interfere throughout life, and the suffering caused by the sexual abuse of children and adolescents becomes even more intense and serious when the victim becomes pregnant with the aggressor, especially in incest situations, being a situation that reveals a double violence and aggression to the rights of children and adolescents.* (Doc 14)

Official documents highlight the need to involve the different public policy management segments of society in the fight against sexual violence against children. They emphasize that the three levels of government are responsible for promoting actions aimed at identifying violence and offering adequate conditions for professionals working in care services, contributing to the fight against institutional violence.

*The union, the states, the Federal District and the cities may periodically promote awareness campaigns for society, promoting the identification of violations of rights and guarantees of children and adolescents and the dissemination of protection services and care flows, as a way to avoid institutional violence.* (Doc 27)

Due to the complexity of sexual violence, they reinforce the need for integration between childcare services, since tackling the problem depends on a structured network for harm reduction, monitoring of victims and periodic assessment of implemented actions.

*Formulate guidelines and parameters for structuring integrated care networks for children and adolescents in situations of violence, based on the principles of celerity, humanization and continuity of care.* (Doc 31)

*The service must be an ethical and professional practice, in accordance with the regulations of the respective professional bodies and cannot aggravate the psychological suffering of child and adolescent victims or witnesses of crimes, and the time and silence of those who are heard must be respected, with emergency protection measures prevailing.* (Doc 30)

In addition to the responsibility of the State and the services that make up the intersectoral network, the participation of the family is mentioned for comprehensive child protection.

(...) *strengthen family competences in relation to comprehensive protection and education in human rights of children and adolescents in the space of family and community coexistence.* (Doc 31)

This empirical category reveals that the Brazilian State recognizes the problem of sexual violence against children and proposes actions to face it. However, what is verified in the concrete reality is the omission of public management in the implementation of these actions, since Violeta reported having suffered sexual violence for four consecutive years.

### Being a Brazilian girl: gender and generational oppressions

Part of the documents addressed, albeit superficially, issues of gender and generation, recognizing the deleterious consequences of the State’s failure to defend children’s rights as a result of sexism and the androcentric vision prevailing in capitalist society. The approach of gender and generation was considered superficial, due to the use of terms without sufficiently describing their concept or supporting them theoretically in the relevant references.

*The International Declaration of Sexual Rights recognizes that the rights of women and girls are a comprehensive part of universal human rights that sexual rights are the inalienable heritage of all human beings and that their promotion and protection is a primary responsibility of governments.* (Doc 20)

*The difficulty in accessing the right and its almost obstacles contributed to prolonging the suffering and reinforcing the violence already suffered by this child and her family. It is a process that explains the strength of patriarchy and oppression over women in the capitalist and sexist society in which we live. Such a situation cannot be made invisible, nor naturalized.* (Doc 19)

Some speeches highlighted that gender inequality conditions women from childhood to a subordinate position in the social hierarchy, which increases the risk of exposure to different types of violence. Thus, State actions aimed at guaranteeing the human rights of girls and women stand out as fundamental. The documents also emphasize the need to address gender inequality from childhood, given that violence is a social construction.

(...) *considering that inequalities between girls and boys are socially and culturally constructed from childhood and adolescence, shaping conceptions and behaviors that often disadvantage girls and lead to violations and violence in their life trajectories related to the fact that they are women.* (Doc 19)

*They underscore the urgent need for gender inequality to be recognized and addressed from childhood and adolescence, especially through policies, budget and public services that include sex education to decide, contraceptives to avoid [having an] abortion and legal abortion to avoid dying.* (Doc 14)

Regarding the generation category, the documents give visibility to advances in the field of children’s rights, from the approval of ECA, with emphasis on the necessary prioritization of actions so that children and adolescents are recognized as social subjects and with rights.

*The vision of the “child-object”, of the “minor child”, i.e., the hygienist and correctional vision is replaced by the vision of children as subjects of rights. The most important part of this movement, inaugurated by the Constituent Child and which culminated in the approval of the Child and Adolescent Statute - ECA, in 1990, is the affirmation of the universality of children’s rights.* (Doc 31)

(...) *considering that the Child and Adolescent Statute - Law 8.069/1990 (ECA) recognizes children and adolescents as people in a peculiar condition of development and as subjects of rights, worthy of receiving full protection and having their best interest guaranteed, it is established that their rights must be promoted and protected in the first place as an absolute priority.* (Doc 14)

In the documents analyzed, a change in the conception of children can be observed, since from ECA they began to be understood as social subjects with rights. However, it appears that Violeta was not treated, in fact, as a bearer of rights, in most of the case. Through her silencing, the aggressor’s threat, the re-victimization by professionals and public agents, the initial devaluation of her desire to terminate her pregnancy and the blame for abortion are identified.

*The girl reportedly said that she had been a victim of the crime since she was 6 years old and that she did not report it out of fear because she was threatened.* (Doc 7)

(...) *that rape be condemned and the rapist be punished and, above all, be understood, once and for all, that it is necessary to STOP blaming girls and women, victims of a heinous crime, which represents the most perverse expression of patriarchy and sexism.* (Doc 12)

The official documents selected in this research did not specifically address child pregnancy, only general issues related to girls’ and adolescents’ sexual and reproductive rights. The discussion on legal abortion was exclusively guided by the Penal Code, considering the procedure from the perspective of criminalization.

## DISCUSSION

Brazilian public policies for childcare are important elements for structuring a system of legal guarantees of rights. This system involves subjects and services that constitute the support network responsible for the prevention, accountability and care of situations of violence^(15)^.

Although the results of this research show advance in the guarantee of children’s rights in Brazil, they also reveal setbacks, especially in the implementation of actions for such realization. Given the diversity of violations to which Violeta was exposed, the question is: to what extent does Brazil protect and guarantee children’s rights? The data from this study show that challenges related to coping with sexual violence and access to legal abortion are still present in Brazilian society. They are associated with the predominant conception of society as androcentric and patriarchal, which establishes a unilateral adult-child power relationship, in which children are considered as property and controlled through silencing and subalternization.

After much discussion and mobilization, especially of women’s movements, responsible for occupying spaces where conservative groups were concentrated for the defense of children’s rights, Violeta’s decision was respected. Historically, feminist movements are the main responsible for discussion about abortion, in the sense that women have autonomy over their bodies and destinies, as well as for public policies on the subject to have legal and institutional advances^(16)^.

The violations experienced by Violeta went beyond the singular sphere and were reproduced in particular by professionals from the hospital service responsible for the first care and by conservative groups that spoke out against abortion and exposed the child’s and their family’s privacy on social networks.

An African study conducted with 86 health professionals working in post-abortion care found that 27% of participants considered the interruption of pregnancy a sin. They also suggested counseling as a measure of care for continuity of pregnancy, even when abortion was legally guaranteed^(17)^. Another study, conducted with two rape victims in India, indicated that they had to resort to the justice system after health professionals refused the procedure. This data revealed the need for training of health professionals for developing good practices, sensitive to gender issues, in order to reduce barriers to access, attitudes of prejudice and neglect^(18)^.

In the structural dimension, there was an influence of political and religious representatives for not interrupting pregnancy, even if supported by law, which reveals the following contradiction: at the same time that the legislation provided support for Violeta to have an abortion, public agents expressed their opposition and objection, solely based on personal beliefs, mostly of a religious nature.

In Brazil, since 2014, a political polarization has been evidenced, in which the conservative political movements of the extreme right have stood out. These movements are situated in the convergence between neoliberalism, neoconservatism and religious fundamentalism. They consider that gender ideology is a threat to the moral values of society, so it needs to be fought. In this context, there is a predominance of discourses that naturalize and justify gender violence as a way of maintaining the current social order^(19)^.

Conservative ideology politically structured has an influence on the institutions of power that make up Brazilian society, such as the justice and health system. In the Western world, power materializes in its own legal language and uses strategies to take root and be exercised in the micro-relationships of power. The use of legal norms by doctors and jurists to deny assistance to women seeking abortion provided by law is a result of an unequal relationship of power, anchored in the growing relevance of regulatory and regulatory actions^(20)^.

Thus, the defense of conservative conceptions, based on religious beliefs and expressed by public agents, to the detriment of children’s rights, clearly reveals the absence of secularism in the Brazilian State. Moreover, the criminalization of abortion stands out, despite the circumstance in which pregnancy occurred^(21)^. According to Brazilian legislation, abortion is allowed when pregnancy represents a risk to a pregnant woman’s life or is a result of sexual violence^(22)^. Violet’s pregnancy met both requirements, but the right to terminate it was not immediately assured after her decision.

The appreciation of an unborn child’s life as opposed to a pregnant child’s life by conservative groups that were against the decision for legal abortion reveals the intersectionality of gender and generation issues. With regard to gender, the sacredness of motherhood was considered more relevant than the situation of sexual violence against a child. In contrast, the sacredness of Violeta’s body, which was violated, was not the subject of an equally intense debate by the same agents who preached the concept’s salvation. In terms of generation, children’s autonomy in decision-making was completely annulled, with adults’ will prevailing even though Violeta had repeatedly stated her desire to terminate her pregnancy.

Respect for children’s desires, when making decisions about their body, is related to care and attention given to listen to them in a qualified listening, which is not only correct, but necessary. Only by listening to it will it be possible to understand the nature, scale and impact of violence on their life. The right to participate, manifest and have their will respected is not only fundamental to children’s dignity and autonomy, but as a vital dimension of protection against the abuse and exploitation of those with less power^(23)^.

The discourses analyzed reveal the domination of aggressors and the different social subjects over childhood and the female body by silencing their rights, desires and autonomy. In view of this, public policies for child protection proved to be insufficient. To ensure the girl’s right to abortion, it was necessary to break with the various conceptions that reinforce the unequal and oppressive character of man-woman and child-adult relations^(24)^.

The superficial approach of gender and generation content in the official documents analyzed proved to be an important obstacle, as it was not possible to know how they were considered. Gender inequality is responsible for female subalternity and is expressed through different types of violations^(25)^. Also, girls are also considered family property and without rights^(26)^.

Recognizing children as subjects of rights has advanced in the last 40 years, mainly through the Convention on the Rights of the Child, held in 1989^(23)^, which had an impact on the legislation of different countries. In Brazil, although part of the official documents reflects this change of conception, this was not confirmed in the case analyzed.

The situation of sexual violence became evident when Violeta was already pregnant. Fear of her uncle’s threats prevented her from seeking support from the formal and informal support network. Their silence can also be linked to blaming and accountability for situations of violence, as girls are continuously judged by their behaviors, ways of speaking, dressing and playing^(27)^, which reinforces the culture of rape, widespread in society.

The understanding of violence as a product of power relations established between adults and children contributes to deconstructing the rape culture that reifies and eroticizes the bodies of girls for the sexual satisfaction of adult men^(28)^. The recognition of violence against children as a result of a historical and social construction is essential to prevent, identify and address the problem.

The findings of this research confirm that children, especially girls, form a vulnerable social group, subject to different types of violations that go beyond the family sphere and reproduce in the public spaces of society. Therefore, investment in an efficient and effective support network is fundamental to guarantee children’s rights.

The responsibility of the family, civil society and State in protecting children and in confronting the violence of which they are victims reveals the complexity of the phenomenon and the urgency of concrete actions in defense of children protection. The comprehensive children protection depends on an integrated network, consisting of qualified professionals and with adequate conditions of care, to identify and intervene in situations of violence^(29)^. Furthermore, children need to have a voice and listening space so that their demands are transformed into effective public policies.

It is necessary to denaturalize violence in society and to overcome the barriers that prevent education for emancipation. A study conducted in Vietnam with schoolchildren identified that they did not receive sufficient information about sexuality from parents and the school, resulting in distorted perceptions about sexual abuse, such as that perpetrators would not be related or that school and home were safe places^(30)^.

Cases of violence against children and adolescents require the strengthening of services and actions to meet their needs, which should include, from adequate treatment by justice and public security systems, to physical, emotional and psychosocial support offered by health systems, social assistance and education^(31)^. However, the structuring of child protection services, the lack of investments and conservative political-ideological positions in relation to the topic make it difficult to apply protective legislation.

Therefore, in addition to the restructuring of health services and welcoming, it is also necessary to combat the interference of religious groups and private interests in the Brazilian State. It is essential that governments cease to invoke religious customs, traditions or influences to avoid fulfilling their obligations in protecting rights and enter into a commitment to condemn violence against women and children^(32)^.

### Study limitations

The present study had as limitation the access only to official documents published online and publicly available. The data collection period, covering only the months in which the case was disclosed, was also a limitation due to Violeta’s entry into the Victims and Witnesses Protection Program, given the repercussion of the case and the threats made to her. However, these limitations do not invalidate the results, as the data were sufficient to highlight the contradictions and support the analysis of the phenomenon.

### Contributions to public policies

The results of this study add new knowledge about sexual violence, child pregnancy and legal abortion, drawing attention to a phenomenon still little explored in public policies, as well as in scientific literature. In addition to this, they raise reflections on the Brazilian system of guaranteeing rights, the implementation of public policies and the strengthening of care for girls who are victims of sexual violence.

## FINAL CONSIDERATIONS

The study of ideological perspectives contained in documents and official discourses on the subject revealed that, although they contained concepts related to respect for children’s rights and child protection, these were not enough to recognize and confront sexual violence, child pregnancy and access to legal abortion, in this specific case.

The official documents analyzed revealed conservative conceptions as the main barrier to guaranteeing children’s rights, based mainly on religion and the principled criminalization of abortion. These conceptions, when expressed by political representatives, also call into question the Brazilian State secularism, in spite of having declared itself so, since the Federal Constitution of 1988.

In addition, the superficiality with which gender and generation contents were addressed in official documents reveals the need to deepen and spread these concepts, as well as their contextualization, so that the phenomenon of violence can be understood and faced from a critical and emancipatory perspective.

Finally, it is necessary to combat and overcome androcentrism and patriarchy within governmental bodies, allowing women and children to also occupy the spaces for debates and decisions. Moreover, it is urgent to recognize that the support network for children’s rights requires financial investments from managers of the three spheres of government, to enable the improvement of tools, strategies and critical positions to face violence. The underfunding of actions that is now evident reveals a perverse facet of the current scenario of setback and dismantling of current public programs and policies.

The study also showed the strength of popular movements - especially women - in the search for the guarantee of rights of children involved. Thus, it is necessary to recognize and enhance this action, with the health sector allied to social control in favor of the defense of sexual and reproductive rights of people of all generations.
